# Nutritional Management in Cirrhosis and Hepatic Encephalopathy: Current Practices and Expert Opinions of Indian Gastroenterologists

**DOI:** 10.7759/cureus.113933

**Published:** 2026-08-04

**Authors:** Nagaraja R Padaki, Kr Vinayakumar, Jyoti Mohapatra, Akash Roy, Abhishek Banerjee, Karan Kumar, Sourabh B Sonawale, Nandan Joshi

**Affiliations:** 1 Department of Gastroenterology, Asian Institute of Gastroenterology, Hyderabad, IND; 2 Department of Gastroenterology, Ananthapuri Hospitals and Research Institute, Thiruvananthapuram, IND; 3 Department of Gastroenterology, Peerless Hospital, Kolkata, IND; 4 Department of Gastroenterology, Apollo Multispeciality Hospitals, Kolkata, IND; 5 Department of Gastroenterology, Manipal Hospitals, Kolkata, IND; 6 Department of Gastroenterology, Mahatma Gandhi Medical College & Hospital, Jaipur, IND; 7 Department of Medical and Scientific Affairs, Dr. Reddy's and Nestlé Health Science Limited, Hyderabad, IND; 8 Department of Medical and Scientific Affairs, Dr. Reddy’s and Nestlé Health Science Limited, Hyderabad, IND

**Keywords:** branched-chain amino acids, chronic liver disease, hepatic encephalopathy, malnutrition, sarcopenia

## Abstract

Background: Chronic liver disease (CLD) is increasingly prevalent in India and is frequently associated with malnutrition, sarcopenia, and hepatic encephalopathy (HE). Although national and international guidelines emphasize adequate nutrition and protein intake, real-world clinical practices in the Indian setting remain insufficiently characterized.

Methods: A cross-sectional descriptive analysis comprising 12 questions was conducted using live polling and moderated discussions across six advisory board meetings over five months. A total of 57 gastroenterologists from across India participated. Discussions focused on compensated and decompensated cirrhosis and HE. Polling responses were analysed question-wise because participation varied across items.

Results: Half of the clinicians assessed nutritional status every three months, while one-third relied on symptom-triggered evaluation. Lack of screening tools (39.6%) and limited awareness (29.2%) were major barriers to early diagnosis. Nutritional supplementation was primarily initiated based on sarcopenia (63.6%). High-protein supplements were recommended to at least half of CLD patients by most clinicians, with 37.2% prescribing them to 50-75% of patients. As disease severity progressed, 56.8% increased protein intake to ~1.5 g/kg/day. For protein intolerance, oral branched-chain amino acids (BCAAs) were preferred (51.2%). In HE, clinicians favoured maintaining protein intake with BCAA addition rather than restriction, and muscle preservation was the most recognized benefit (80%). In transplant candidates, a high-protein diet (~1.5 g/kg/day) with adjunct BCAA supplementation was prioritized. Poor taste, gastrointestinal intolerance, and cost were key adherence barriers.

Conclusion: Overall, the findings indicate an increasing shift toward proactive nutritional management in CLD within Indian clinical practice, with routine screening commonly practiced, and 56.8% of clinicians reporting escalation of protein intake from 1.2 to 1.5 g/kg/day as disease severity progressed. BCAA supplementation was also considered, particularly in hepatic encephalopathy and advanced disease, to support muscle preservation and clinical outcomes.

## Introduction

Chronic liver disease (CLD) refers to persistent impairment of hepatic structure and function lasting longer than six months. It is marked by progressive loss of synthetic capacity, including reduced production of clotting factors and essential proteins, impaired detoxification of metabolic by-products, and altered bile excretion. Pathophysiologically, CLD involves ongoing inflammation, hepatocellular injury, and regeneration, eventually leading to fibrosis and cirrhosis [1].

In India, CLD has emerged as a significant public health concern, driven by rapid urbanization, dietary transitions, and changing alcohol consumption patterns [2]. The etiological spectrum is now dominated by metabolic dysfunction-associated steatotic liver disease (MASLD, formerly non-alcoholic fatty liver disease (NAFLD)), including metabolic dysfunction-associated steatohepatitis (MASH), and alcohol-associated liver disease (ALD) [2]. The reported prevalence of MASLD varies widely (2.6-43.6%), reflecting population heterogeneity but highlighting the growing burden [3,4]. Cirrhosis remains a leading cause of mortality, accounting for approximately 22.2 deaths per 100,000 population and ranking among the top 10 causes of death in India [2].

As CLD progresses to decompensation, complications such as hepatic encephalopathy (HE) may develop. The failing liver is unable to effectively clear neurotoxins like ammonia, resulting in their accumulation and subsequent neurological dysfunction. Concurrently, malnutrition frequently develops and acts both as a consequence and a driver of disease progression. It is strongly associated with increased complications, worsening liver function, and reduced survival, particularly in decompensated cirrhosis [1].

Given this interplay, nutritional assessment is critical in chronic liver disease management. Commonly used tools include the Subjective Global Assessment (SGA), Patient-Generated Subjective Global Assessment (PG-SGA), and Global Leadership Initiative on Malnutrition (GLIM) criteria [5]. Early identification of malnutrition enables timely intervention, which can reduce complications, improve quality of life, and lower mortality risk. Patients with cirrhosis require adequate caloric and protein intake to prevent muscle catabolism and maintain metabolic balance. Current recommendations suggest 30-35 kcal/kg/day (based on dry body weight), with 50-60% carbohydrates, 20-30% fats, and 1.2-1.5 g protein/kg/day [5-7]. Dividing intake into multiple meals, including a late-evening carbohydrate-rich snack, is also advised to minimize prolonged fasting and muscle breakdown [6].

Protein restriction is no longer recommended in patients with HE. Instead, adequate protein intake, preferably from vegetable and dairy sources, should be maintained. In cases of intolerance, branched-chain amino acids (BCAAs) may be used, offering benefits in managing HE and improving muscle mass and functional outcomes [8].

Despite these advances, India-specific expert guidance remains limited, highlighting the need to better understand real-world nutritional practices in CLD. This expert opinion, therefore, explores challenges in diagnosing and managing malnutrition and reviews nutritional strategies to improve clinical outcomes.

## Materials and methods

Study design

This expert opinion is based on a cross-sectional descriptive analysis of live electronic polling responses and moderated expert discussions conducted during six advisory board meetings, from September 2024 to May 2025. The advisory boards focused on contemporary clinical practices related to the nutritional management of patients with compensated and decompensated cirrhosis, including hepatic encephalopathy.

Study participants

A total of 57 practicing gastroenterologists from different regions of India participated in the advisory board meetings. All participants fulfilled the predefined eligibility criteria and were included in the final analysis.

Inclusion and exclusion criteria

Eligible participants were practicing gastroenterologists or hepatologists from across India, actively involved in the management of patients with chronic liver disease, with at least five years of independent clinical experience in this field. Participants were required to have attended at least one advisory board meeting and provided informed consent for participation in the live polling exercise and moderated scientific discussions. Healthcare professionals from specialties other than gastroenterology or hepatology, individuals who declined to provide informed consent, and participants who did not respond to any polling question during the advisory board meetings were excluded from the analysis.

Sampling method

Participants were recruited using purposive sampling to ensure representation of experienced gastroenterologists from different geographical regions of India. The objective was to obtain expert perspectives on current nutritional practices in CLD rather than generate nationally representative estimates.

Sample size

As this study represents an advisory board-based expert opinion exercise, no formal sample size calculation was performed. The analytical sample was determined by the number of eligible experts who attended the advisory board meetings and consented to participate. Consequently, all eligible participants were included in the analysis.

Questionnaire development

A structured questionnaire comprising 12 multiple-choice questions was developed collaboratively by the authors based on current scientific evidence, international clinical practice guidelines, and the predefined objectives of the advisory board meetings. The questionnaire was designed to generate both quantitative polling responses and qualitative expert insights through moderated scientific discussions. It explored the entire spectrum of nutritional management in CLD, including current practices in nutritional screening and assessment, recommended protein requirements according to international guidelines, real-world protein intake practices, barriers to achieving optimal nutritional goals, the role and selection of oral nutritional supplements, management of protein intolerance, use of BCAAs, nutritional management in hepatic encephalopathy, and nutritional strategies before and after liver transplantation. In addition, the discussions explored the practical challenges faced in routine clinical practice and potential strategies to improve implementation of evidence-based nutritional care. Before administration, the questionnaire was reviewed by the expert faculty for content relevance and clarity. As its purpose was to facilitate structured scientific discussions during the advisory board meetings, it was not designed or validated as a psychometric survey instrument. The complete questionnaire is provided in the Appendices.

Data collection

Before the commencement of each advisory board meeting, participants were informed about the objectives of the meeting, the polling process, and the intended use of anonymized responses for scientific publication. Informed consent was obtained from all participants before participation. The questionnaire was administered using live electronic polling before the moderated scientific discussions to minimize the influence of peer interactions on individual responses. Responses were collected anonymously in real time, and aggregated data were used for analysis.

Endpoints

Primary Endpoint

The primary endpoint of this expert opinion study was to characterize current clinical practices and identify key challenges in the diagnosis, nutritional assessment, and management of malnutrition in patients with compensated and decompensated cirrhosis, including hepatic encephalopathy, based on anonymized responses obtained using live electronic polling of experienced gastroenterologists and hepatologists.

Secondary Endpoints

The secondary endpoints were to evaluate expert perspectives on nutritional screening and assessment practices, recommended protein requirements and real-world protein intake, the use of oral nutritional supplements and branched-chain amino acid supplementation, approaches to managing protein intolerance and HE, nutritional strategies before and after liver transplantation, and perceived barriers to implementing evidence-based nutritional recommendations in routine clinical practice. Additionally, the study sought to summarize areas of consensus and variation in expert opinion regarding nutritional management across the spectrum of chronic liver disease.

Statistical analysis

Data were analyzed using descriptive statistics. Frequencies and percentages were calculated for each response option based on the number of participants responding to the respective question. As participation varied across individual polling questions, the denominator differed accordingly and is reported for each figure. No inferential statistical analyses were performed because the objective of the study was to describe expert opinions and current clinical practice patterns rather than test predefined hypotheses.

## Results

Current practices in nutritional assessment

Malnutrition and sarcopenia are highly prevalent in CLD, especially in advanced stages, affecting ~30-70% of patients [9]. Sarcopenia, a key manifestation, is seen in up to 60% of cirrhotic patients and correlates with disease severity (Child-Turcotte-Pugh score), contributing to complications such as HE and poorer survival [10,11].

Given the strong link between nutrition and prognosis, the European Society for Clinical Nutrition and Metabolism (ESPEN) recommends routine nutritional assessment in all patients with liver disease, including evaluation of body composition, sarcopenia, and energy expenditure [10]. GLIM proposes a two-step approach: risk screening (e.g., Nutritional Risk Screening 2002 (NRS-2002)) followed by diagnostic confirmation. Malnutrition is diagnosed when one or more phenotypic criterion (weight loss, low body mass index, or reduced muscle mass) coexists with one or more etiologic criterion (reduced intake or inflammation/disease burden) [12]. Serum albumin may serve as a supportive marker, with levels <35 g/L indicating hypoproteinemia. This has been summarized in Table 1.

**Table 1:** Guidelines and recommendations for nutritional assessment AASLD, American Association for the Study of Liver Diseases; EASL, European Association for the Study of the Liver; ESPEN, European Society for Clinical Nutrition and Metabolism

Guidelines	Recommendations
ESPEN practical guideline: clinical nutrition in liver disease [10]	All cirrhosis patients must undergo routine nutritional screening. Screening should be repeated regularly during follow-up.
2021 Practice Guidance by the American Association for the Study of Liver Diseases [13]	Assessing and reassessing frailty and/or sarcopenia using standardized tools
EASL Clinical Practice Guidelines on nutrition in chronic liver disease [6]	Nutritional assessment should be performed every one to six months in outpatient care and conducted at hospital admission with regular reassessments during inpatient stays.

Expert Panel Interpretation

**Figure 1:**
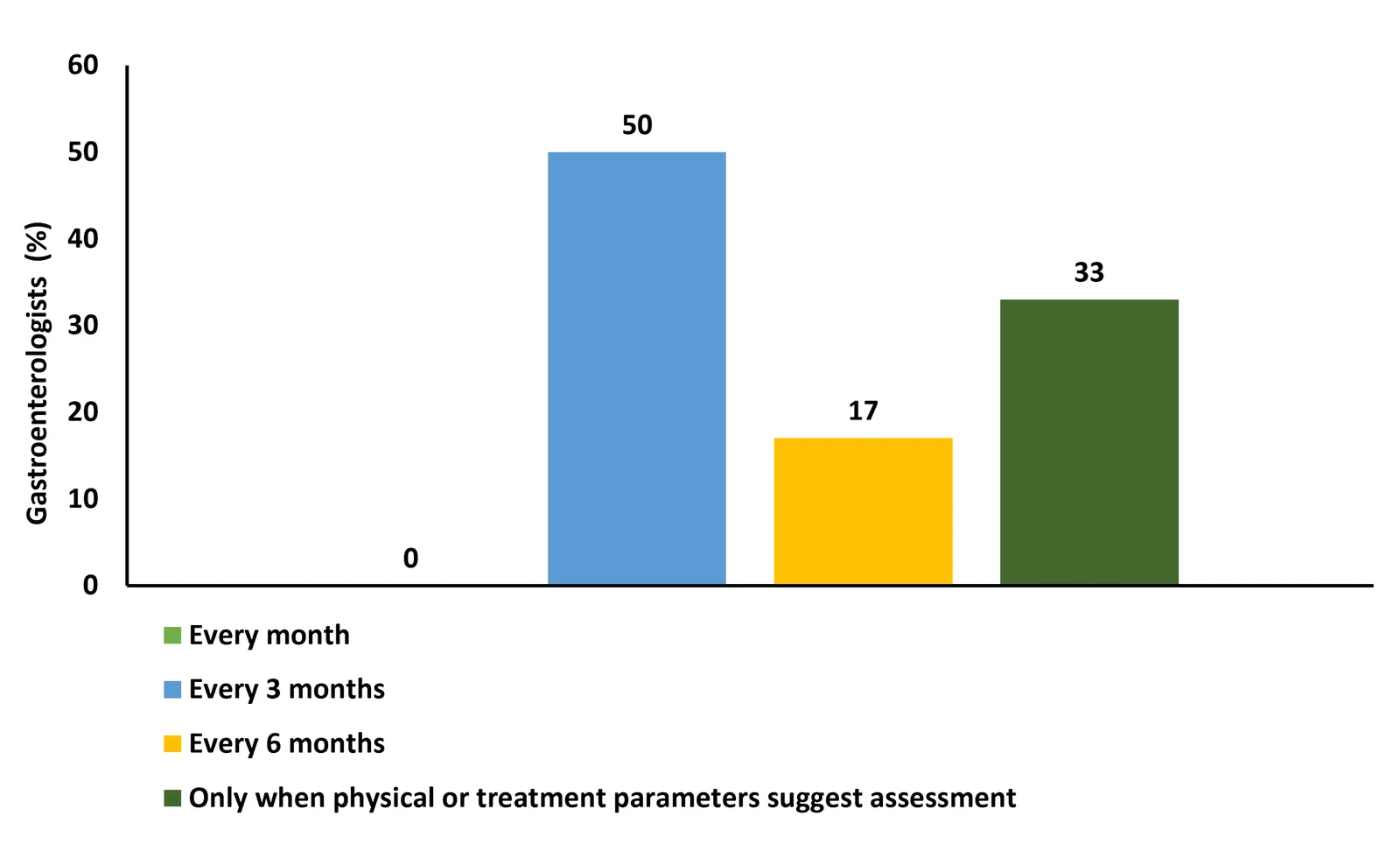
Frequency of nutritional assessment in CLD patients CLD, chronic liver disease

**Figure 2:**
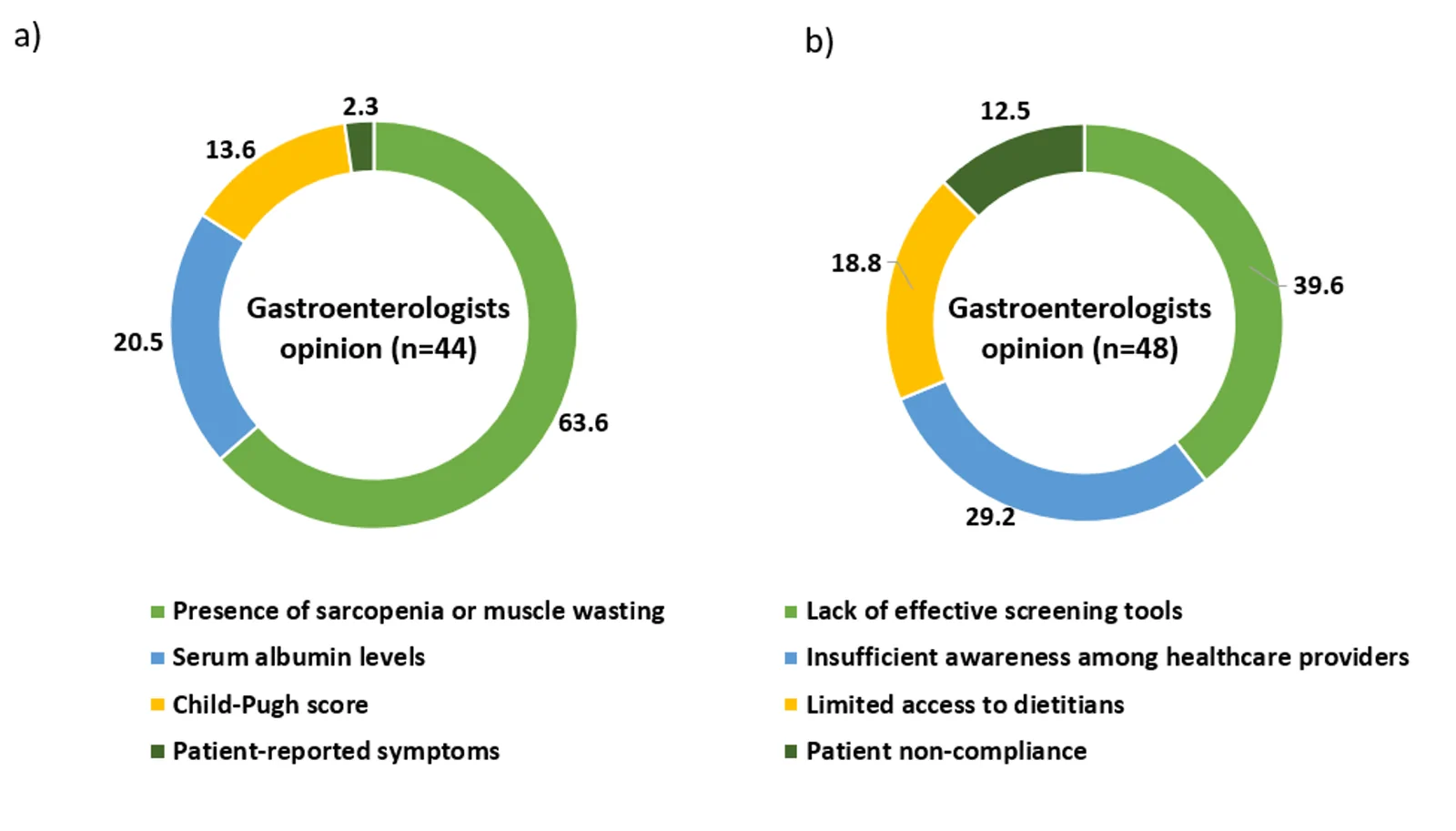
(a) Parameters to rely on while starting nutritional supplements in CLD patients. (b) Primary barriers to early diagnosis CLD, chronic liver disease

A total of 22 (50%) gastroenterologists reported assessing nutritional status in patients with chronic liver disease every three months, making it the most common practice. Approximately 15 (33%) performed assessments only when clinically indicated, reflecting a reactive approach, while 7 (17%) assessed patients every six months. No respondents (0 (0%)) reported conducting monthly nutritional assessments (n=44, Figure 1). Regarding initiation of nutritional supplementation, the majority of respondents (28 (63.6%)) relied on the presence of sarcopenia or muscle wasting, indicating a preference for direct markers of nutritional decline. Approximately 9 (20.5%) based their decision on serum albumin levels, while 6 (13.6%) used the Child-Pugh score. Only 1 (2.3%) initiated supplementation based on patient-reported symptoms (n=44, Figure 2a). The most commonly reported barrier to early diagnosis was the lack of effective screening tools (19 (39.6%)), highlighting a structural gap in routine assessment. This was followed by insufficient awareness among healthcare providers (14 (29.2%)). Limited access to dietitians was reported by 9 (18.8%) respondents, while patient non-compliance accounted for 6 (12.5%) (n=48, Figure 2b).

Expert Opinion

Nutritional assessment in patients with CLD should be conducted routinely, preferably every three months. Nutritional supplementation should be initiated early when sarcopenia or muscle wasting is identified. Implementation of effective screening tools should be prioritized to overcome barriers to early diagnosis.

Protein supplementation patterns across disease stages

Protein restriction, once commonly prescribed in cirrhosis and HE, is now discouraged as it worsens nutritional status without improving outcomes [14]. Current guidelines recommend an adequate intake of ~30-45 kcal/kg/day and 1.2-1.5 g protein/kg/day [5-7]. Clinical evidence supports this shift. Studies show that a high protein intake improves ammonia levels, mental status, and physical condition, while a low protein intake increases muscle breakdown without any added benefit [14-16]. Overall, protein needs are increased in cirrhosis, and long-term intake <60 g/day should be avoided. When dietary intake is insufficient, oral supplements can help. Whey protein isolate is advantageous due to its high biological value, rich BCAA (especially leucine) content, and good tolerability, even in lactose intolerance [7,13]. Protein intake recommendations are summarized in Table 2.

**Table 2:** Guidelines and recommendations for protein intake AASLD, American Association for the Study of Liver Diseases; EASL, European Association for the Study of the Liver

Guidelines	Recommendations
EASL Clinical Practice Guidelines on nutrition in chronic liver disease [6]	Protein intake 1.2-1.5 g/kg/day to prevent muscle loss
2021 Practice Guidance by the American Association for the Study of Liver Diseases [13]	Adults with cirrhosis should receive 1.2-1.5 g/kg/day of protein, as this intake is considered safe, does not exacerbate hepatic encephalopathy, and helps minimize protein catabolism compared with lower protein intakes. In children with cirrhosis, protein intake of up to 4 g/kg/day has also been shown to be safe.

Expert Panel Interpretation

**Figure 3:**
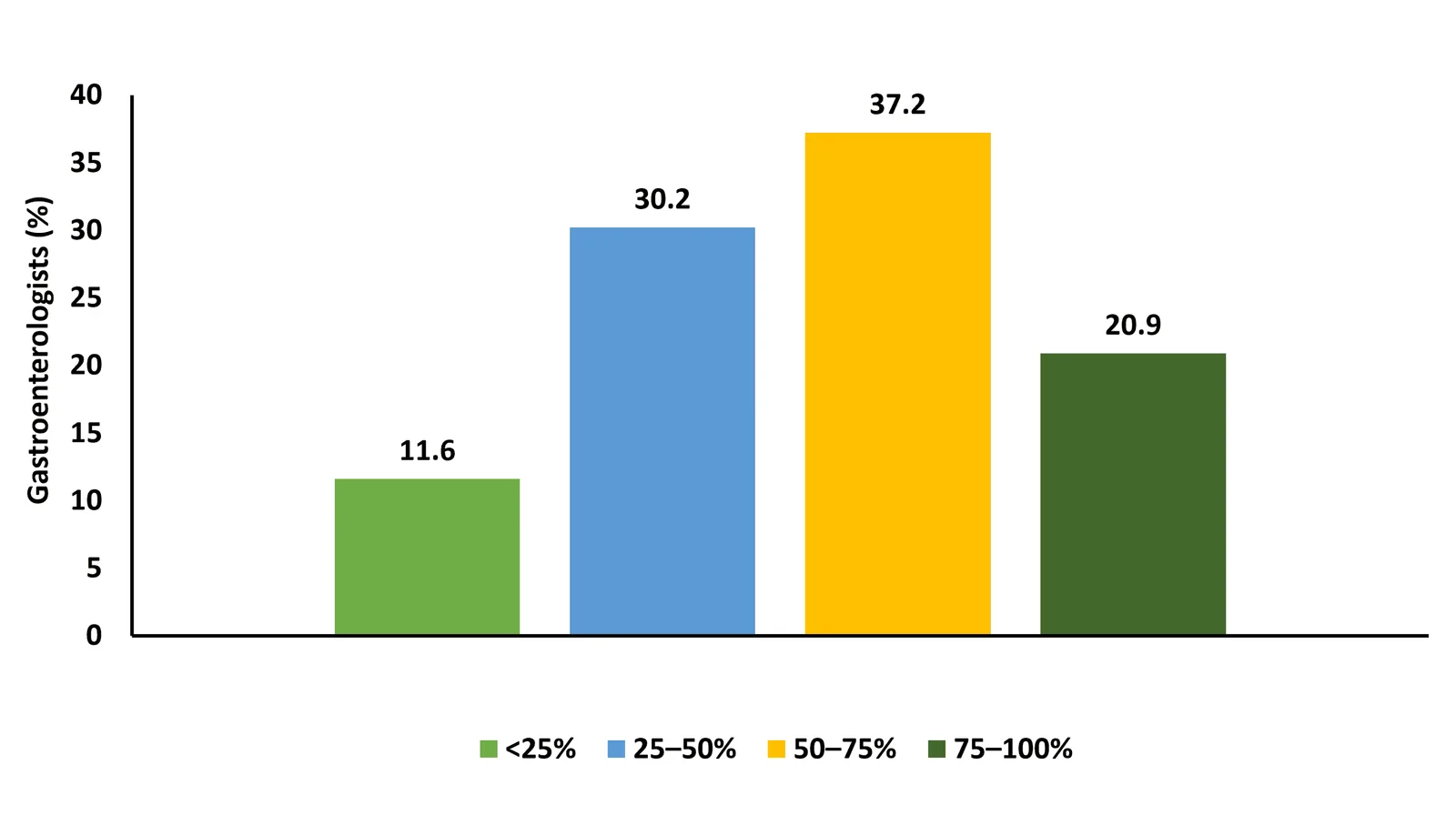
Proportion of CLD patients recommended high-protein supplements CLD, chronic liver disease

**Figure 4:**
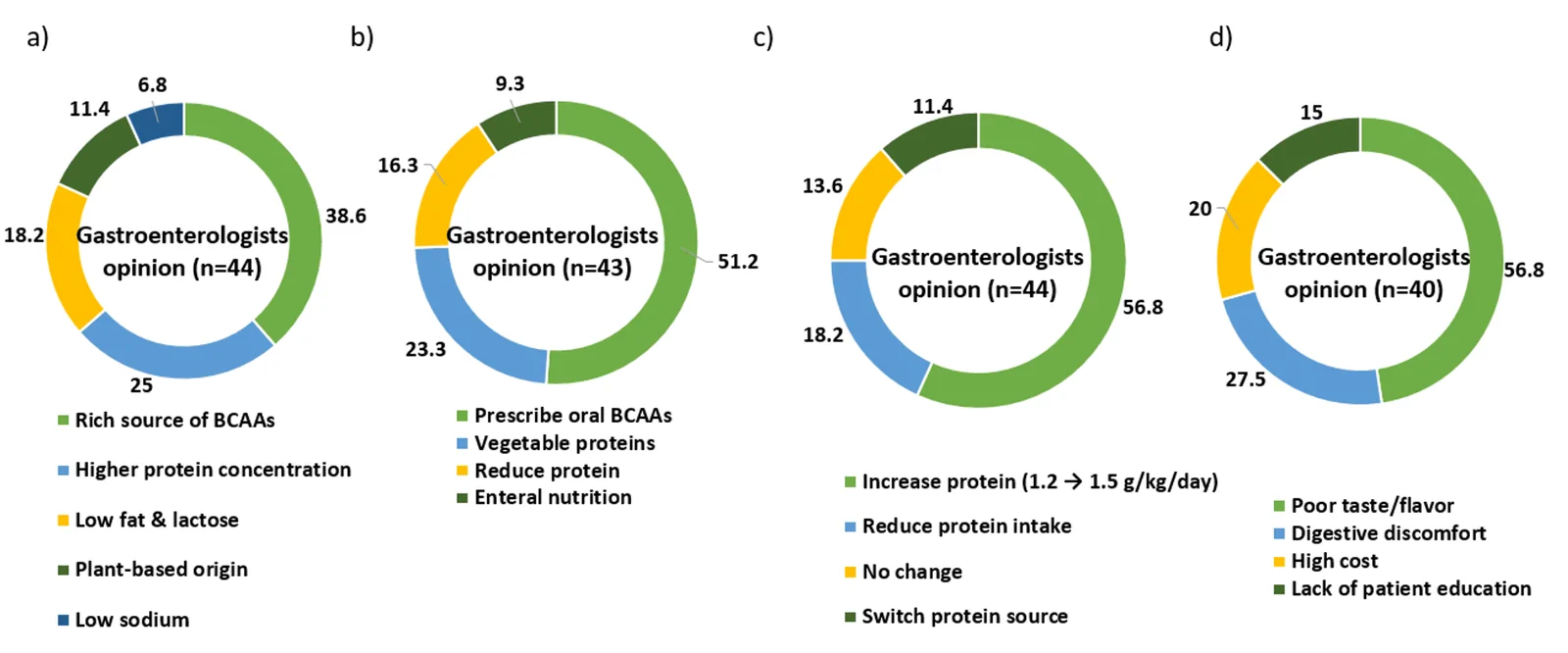
(a) Priorities when selecting the protein source. (b) Approach to protein intolerance. (c) Protein intake strategy, from compensated to decompensated CLD. (d) Challenges in adherence BCAA, branched-chain amino acid; CLD, chronic liver disease

Most gastroenterologists recommended high-protein supplements to a substantial proportion of patients with chronic liver disease, with 16 (37.2%) prescribing them to 50-75% of patients and 13 (30.2%) to 25-50%. Approximately 9 (20.9%) advised supplementation in 75-100% of cases, while only 5 (11.6%) recommended them in fewer than 25% of patients (n=43, Figure 3). When selecting protein sources, the most important factor was a rich BCAA content, reported by 17 (38.6%) respondents, followed by higher protein concentration (11 (25.0%)) and low fat/lactose content (8 (18.2%)). Plant-based origin (5 (11.4%)) and low sodium content (3 (6.8%)) were less frequently prioritized (n=44, Figure 4a). For protein intolerance, over half of the gastroenterologists (22 (51.2%)) preferred oral BCAA supplementation, while 10 (23.3%) opted for vegetable proteins and 7 (16.3%) reduced protein intake; only 4 (9.3%) considered enteral nutrition. This reflects a preference for maintaining protein intake through modification rather than restriction (n=43, Figure 4b). As the disease progressed from compensated to decompensated stages, most clinicians (25 (56.8%)) increased protein intake from 1.2 to 1.5 g/kg/day. Smaller proportions (8 (18.2%)) reduced protein intake, 6 (13.6%) made no change, and 5 (11.4%) switched protein sources (n=44, Figure 4c). The main barriers to supplement adherence were poor taste (23 (56.8%)), digestive discomfort (11 (27.5%)), high cost (8 (20.0%)), and lack of patient education (6 (15.0%)) (n=40, Figure 4d).

Expert Opinion

As patients progress from compensated to decompensated CLD, protein intake should be increased toward ~1.5 g/kg/day and unnecessary restriction should be avoided. High-protein supplements should be used in patients unable to meet requirements through diet alone rather than universally. BCAA-rich, high-concentration protein sources are preferred, and low-fat/lactose formulations are considered when appropriate. In cases of protein intolerance, oral BCAA supplementation should be considered. Improving the palatability of nutritional supplements may enhance long-term adherence. Strategies should also aim to minimize gastrointestinal discomfort, address cost-related barriers, and improve patient education.

BCAA use in HE and advanced cirrhosis

Management of sarcopenia in CLD is based on nutritional optimization and physical activity. BCAA supplementation helps reduce protein catabolism and may improve survival. Isoleucine and valine also aid in lowering ammonia levels by competing with aromatic amino acids at the blood-brain barrier and supporting peripheral ammonia detoxification, thereby potentially improving HE. Accordingly, BCAAs are widely used to preserve muscle mass and support neurocognitive function in CLD [17]. BCAAs leucine, isoleucine, and valine play a key role in muscle metabolism, with leucine being the most abundant and a major driver of muscle protein synthesis via the mechanistic target of rapamycin (mTOR) pathway, thereby helping maintain skeletal muscle mass in cirrhosis and sarcopenia [7,18]. Clinical evidence supports their benefit. A retrospective study (n=2,176) showed that BCAA supplementation reduced cirrhosis-related complications, particularly in viral hepatitis- and alcohol-related cirrhosis [19]. A multicentre study further demonstrated a lower combined risk of HE, ascites, and mortality with BCAA therapy [20]. Recommendations for BCAAs are summarized in Table 3.

**Table 3:** Guidelines and recommendations for BCAAs AASLD, American Association for the Study of Liver Diseases; BCAA, branched-chain amino acid; EN, enteral nutrition; ESPEN, European Society for Clinical Nutrition and Metabolism; HE, hepatic encephalopathy

Guidelines	Recommendations
ESPEN guideline on clinical nutrition in liver disease (2019) [7]	Long-term oral BCAA supplements (0.25 g/kg/day) should be prescribed in patients with advanced cirrhosis to improve event-free survival or quality of life; BCAA-enriched formulas can be used in patients with hepatic encephalopathy in need of EN.
AASLD Practice Guidance for hepatic encephalopathy and malnutrition (2014) [5]	Oral BCAAs can be used as an alternative or additional agent to treat HE patients nonresponsive to conventional therapy; oral BCAA supplementation may allow recommended nitrogen intake to be achieved and maintained in patients intolerant to dietary protein

Expert Panel Interpretation

**Figure 5:**
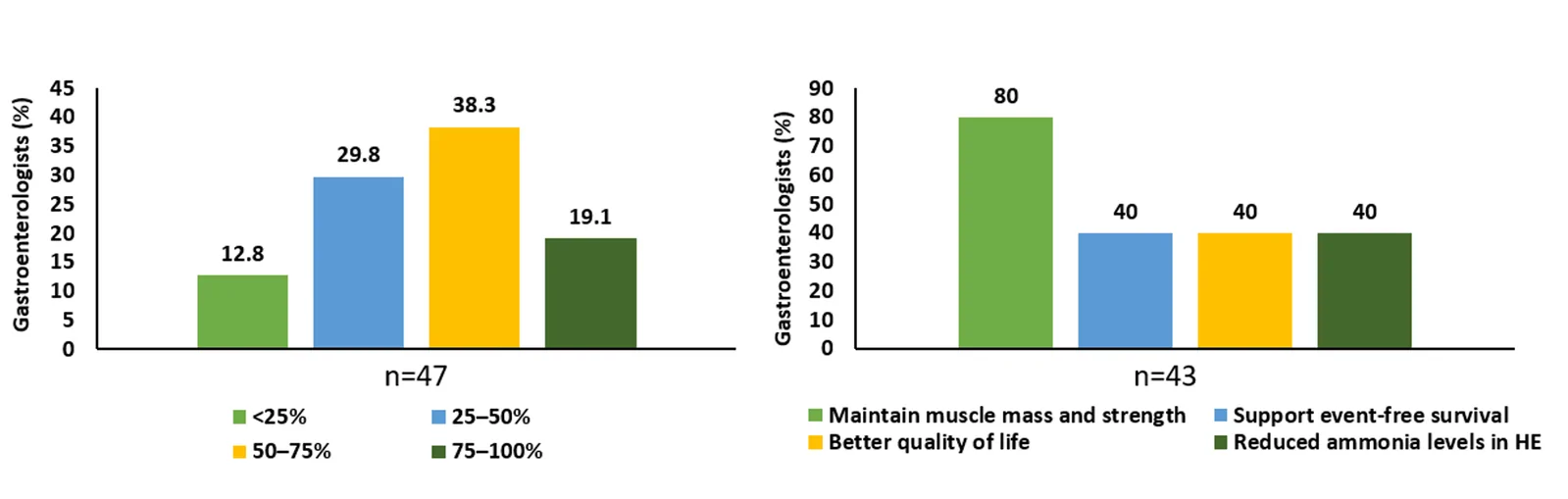
(a) Percentage of HE patients recommended BCAAs. (b) Key benefits of BCAA supplementation in sarcopenia (CLD) patients BCAA, branched-chain amino acid; CLD, chronic liver disease; HE, hepatic encephalopathy

**Figure 6:**
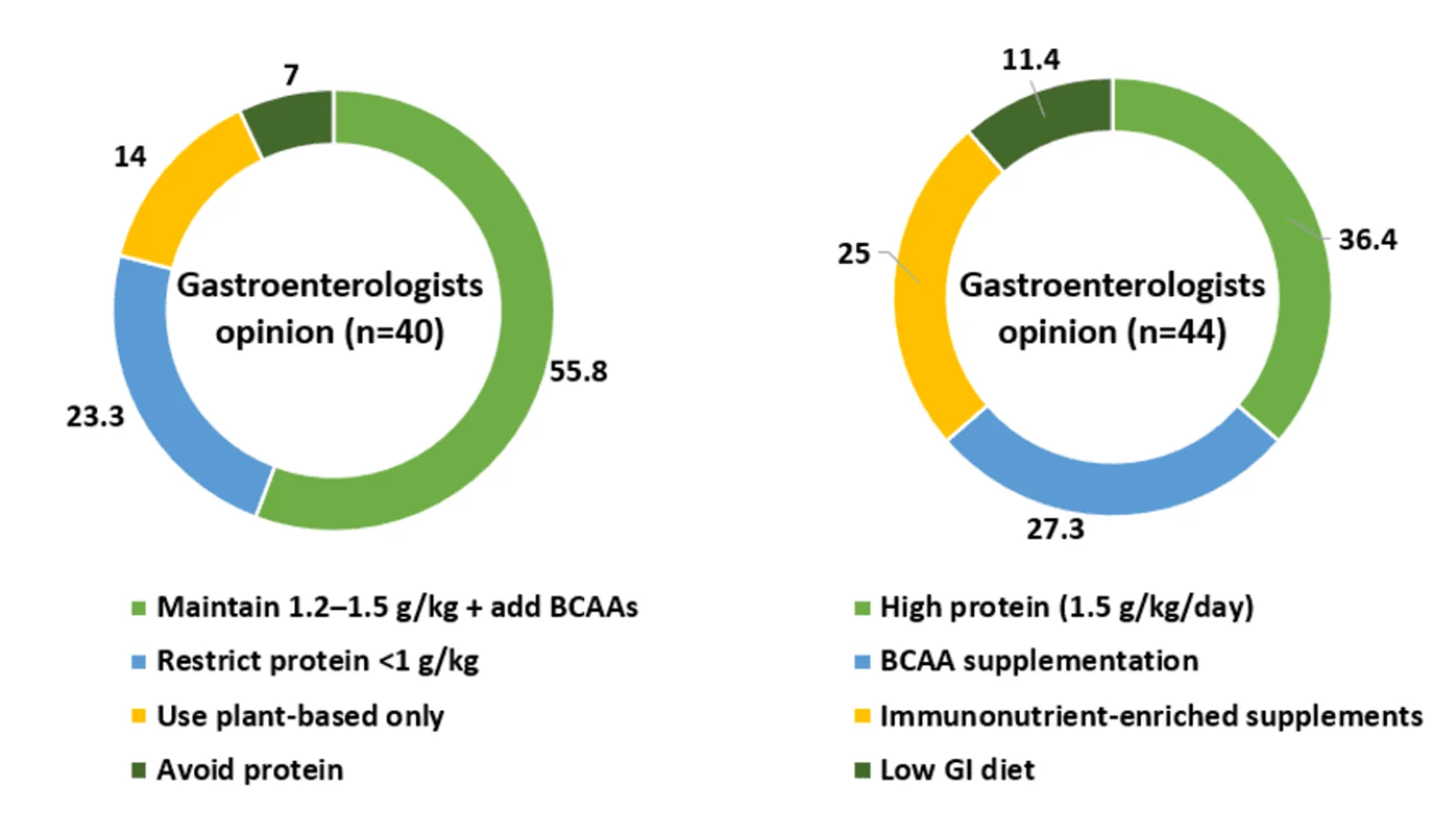
(a) Protein management in HE. (b) Nutritional strategy in pre- and post-transplant patients BCAA, branched-chain amino acid; GI, glycemic index; HE, hepatic encephalopathy

Most gastroenterologists prescribed BCAAs selectively in patients with hepatic encephalopathy rather than universally. The largest group, 18 (38.3%), recommended them to 50-75% of patients, followed by 14 (29.8%) who recommended them to 25-50%. Approximately 9 (19.1%) used them in 75-100% of patients, while 6 (12.8%) prescribed them in fewer than 25%, indicating a tailored approach based on patient profile and disease severity (n=47, Figure 5a). For maintaining protein intake in hepatic encephalopathy, over half of the respondents (22 (55.8%)) preferred continuing adequate protein intake (1.2-1.5 g/kg/day) with BCAA supplementation, reflecting a shift toward preservation rather than restriction. In contrast, 9 (23.3%) still restricted protein intake to <1 g/kg/day, 6 (14.0%) preferred plant-based protein sources only, and 3 (7.0%) advised complete protein avoidance (n=40, Figure 6a). Regarding benefits, the primary perceived advantage of BCAA supplementation was maintenance of muscle mass and strength, reported by 34 (80.0%) respondents. Additional benefits, including improved event-free survival, better quality of life, and reduced ammonia levels in hepatic encephalopathy, were each reported by 17 (40.0%) respondents (n=43, Figure 5b).

Expert Opinion

BCAA supplementation should be selectively integrated into HE management while maintaining adequate protein intake (1.2-1.5 g/kg/day), primarily to preserve muscle mass and strength, with additional benefits including improved survival outcomes, enhanced quality of life, and ammonia reduction.

Nutritional strategy in transplant candidates

Patients awaiting liver transplantation often have significant nutritional deficits that increase perioperative risk, making early nutritional intervention essential [21]. Pre-transplant care focuses on correcting deficiencies and preventing further muscle loss and sarcopenia through adequate intake of protein, energy, fats, vitamins, and minerals, while avoiding complications such as ascites or hyperglycemia [22]. A protein intake of 1.2-1.5 g/kg/day is recommended to preserve muscle mass [23]. Post-transplant, nutritional support remains critical, as patients frequently experience eating difficulties. During rehabilitation, protein intake becomes particularly important for recovery and reducing complications [24]. Evidence shows that energy and protein requirements are often unmet in the first month after transplantation [25,26]. Additionally, persistent nitrogen loss and negative nitrogen balance necessitate increased protein or amino acid intake in the post-transplant period [6]. Nutritional recommendations are summarized in Table 4.

**Table 4:** Guidelines and recommendations for transplant patient EASL, European Association for the Study of the Liver; ESPEN, European Society for Clinical Nutrition and Metabolism

Guidelines	Recommendations
ESPEN peri-operative nutrition guidelines [25]	Protein recommendation ≈1.5 g/kg/day after major surgery, including organ transplantation
EASL Clinical Practice Guidelines on nutrition in chronic liver disease [6]	Nutritional support before transplantation should provide 30-35 kcal/kg/day and 1.2-1.5 g protein/kg/day, depending on whether the goal is maintenance or improvement of nutritional status. After transplantation, 35 kcal/kg/day and 1.5 g protein/kg/day are recommended. In obese patients, enteral and/or parenteral nutrition with 25 kcal/kg/day and 2.0 g protein/kg/day may be considered.

Expert Panel Interpretation

The most prioritized nutritional strategy in the pre- and post-transplant setting was a high-protein diet (1.5 g/kg/day), selected by 16 (36.4%) gastroenterologists. This was followed by branched-chain amino acid supplementation, preferred by 12 (27.3%) respondents, and immunonutrient-enriched supplements, chosen by 11 (25.0%). Only 5 (11.4%) respondents prioritized a low-glycemic index diet. These nutritional strategies are summarized in Figure 6b (n=44).

Expert Opinion

In the pre- and post-transplant period, high protein intake (~1.5 g/kg/day) should be prioritized, supported by BCAAs and immunonutrient supplementation to optimize nutritional reserves and recovery.

## Discussion

Key clinical insights from expert opinions

The expert opinions provide important insights into the real-world nutritional management of chronic liver disease in India, extending beyond existing guideline recommendations. The findings demonstrate a clear shift in clinical practice from protein restriction toward proactive nutritional optimization. Most clinicians reported maintaining or increasing protein intake with disease progression, with over half escalating targets from ~1.2 to ~1.5 g/kg/day in advanced stages. Sarcopenia emerged as the primary trigger for intervention, with clinicians relying more on muscle wasting than biochemical markers, reflecting increasing recognition of skeletal muscle as a key therapeutic target. Despite this progress, gaps remain in routine practice. Approximately one-third of clinicians assess nutritional status only when clinically indicated, indicating that systematic screening is not yet fully integrated. BCAAs are used selectively, primarily to maintain nitrogen balance in patients with HE or protein intolerance while preserving muscle mass. Several practical barriers to implementation were identified, including a lack of standardized screening tools, limited access to dietitians, gastrointestinal intolerance, poor palatability of supplements, and cost constraints. These findings highlight current practice patterns and emphasize opportunities to strengthen the implementation of guideline-recommended nutritional care in Indian patients with cirrhosis.

Summary of recommendations

Routine nutritional assessment and early identification of sarcopenia should be prioritized in CLD management. Protein intake should be optimized (~1.5 g/kg/day), with emphasis on high-quality, BCAA-rich, and well-tolerated protein sources. In patients with intolerance, alternative strategies such as BCAA supplementation, whey protein isolate, or vegetable proteins may be considered. Nutritional management should also address adherence barriers and ensure adequate support during pre- and post-transplant care.

Limitations

This expert opinion has several limitations. Participants were invited clinicians, introducing potential selection bias and limiting generalizability across all practice settings in India. Responses were collected through live polling and moderated discussions, which may be influenced by facilitator and response bias, and therefore reflect reported rather than actual clinical practice. Not all participants answered every question, resulting in variable denominators across analyses. In addition, the questionnaire consisted of non-validated, multiple-choice items designed for expert opinion-building rather than serving as a formal survey instrument. Finally, as a cross-sectional observational exercise, the findings describe prevailing opinions and practice patterns but cannot establish causality or clinical impact. Additional limitations of this study include the limited national representativeness of the expert panel, the potential for commercial or advisory board-related influence, the lack of independent validation of self-reported clinical practices, and the absence of a systematic qualitative analysis of the moderated discussions.

Future research scope

Future studies should evaluate the real-world implementation of standardized nutritional protocols and their impact on clinical outcomes such as sarcopenia progression, hospitalization, and hepatic encephalopathy recurrence. Prospective multicenter studies with validated assessment tools are needed to confirm practice patterns identified in this expert opinion. Additionally, research assessing the effectiveness of structured clinician training programs and multidisciplinary nutritional care models, including dietitian-led interventions, may help determine strategies that improve adherence to guideline-recommended nutritional management in chronic liver disease.

## Conclusions

This analysis highlights a transition in Indian clinical practice toward proactive and individualized nutritional management in CLD. Increasing emphasis is being placed on adequate protein intake and early intervention to mitigate sarcopenia and its associated complications. The findings underscore the need for structured nutritional screening, standardized treatment approaches, and improved integration of nutritional care into routine clinical practice. Strengthening clinician awareness and addressing practical barriers may further enhance the adoption of evidence-based nutritional strategies and improve patient outcomes.

## Appendices

**Table 5:** Questionnaire CLD, chronic liver disease; BCAA, branched-chain amino acid; GI, glycemic index

Poll Question	Response Options
How often do you assess the nutritional status of your CLD patients to monitor malnutrition risk?	Every month, Every 3 months, Every 6 months, Only when clinically indicated
Which parameter do you primarily rely on to start nutritional supplements in CLD patients?	Child-Pugh score, Presence of sarcopenia, Serum albumin, Patient-reported symptoms, Other
What is the primary barrier to early diagnosis of malnutrition in CLD patients? (Select top 2)	Lack of screening tools, Insufficient awareness, Patient non-compliance, Limited access to dietitians, Other
What percentage of your CLD patients do you recommend liver-specific or high-protein supplements for?	<25%, 25-50%, 50-75%, 75-100%
How does progression from compensated to decompensated cirrhosis influence your protein supplementation strategy?	Increase to 1.5 g/kg/day, Switch protein source, Reduce protein, No change
What is your primary approach to addressing protein intolerance in advanced cirrhosis?	Vegetable proteins, Oral BCAAs, Reduce protein, Enteral nutrition, No intolerance
What is the most significant challenge in ensuring adherence to protein-rich diets? (Select top 2)	Digestive discomfort, Poor taste, Lack of education, High cost, Other
Which factor do you prioritize most regarding protein quality? (Select top 3)	Higher protein concentration, Low fat and lactose, Rich in BCAA, Plant-based, Low sodium, Taste
What percentage of your hepatic encephalopathy patients do you recommend BCAA supplementation for?	<25%, 25-50%, 50-75%, 75-100%
In HE patients, what is your primary approach to balancing protein intake and ammonia levels?	Maintain 1.2-1.5 g/kg/day + BCAA, Restrict protein, Plant protein only, Avoid supplementation
What benefit have you observed with BCAA supplementation in sarcopenic CLD patients? (Select top 2)	Maintain muscle mass, Event-free survival, Better quality of life, Reduced ammonia
For CLD patients awaiting liver transplantation, which nutritional intervention do you prioritize? (Select top 2)	High protein, BCAA, Immunonutrient supplements, Low-GI diet
